# Facial Emotion Recognition and Polymorphisms of Dopaminergic Pathway Genes in Children with ASD

**DOI:** 10.1155/2020/6376842

**Published:** 2020-11-04

**Authors:** Zhuo Liu, Jun Liu, Zengyu Zhang, Hong Yu, Fengpei Hu

**Affiliations:** ^1^Department of Internal Medicine, Zhejiang Xiaoshan Hospital, Hangzhou, China; ^2^Central Laboratory, Department of Clinical Laboratory, Zhejiang Xiaoshan Hospital, Hangzhou, China; ^3^Department of Pediatrics, Xiaoshan First Affiliated Hospital of Hangzhou Normal University, Hangzhou, China; ^4^Department of Clinical Psychology, Xiaoshan First Affiliated Hospital of Hangzhou Normal University, Hangzhou, China; ^5^Institute of Brain and Management Science, Hangzhou, China; ^6^Department of Psychology, Zhejiang University of Technology, Hangzhou, China

## Abstract

**Background:**

It is inconclusive whether children with autism spectrum disorder (ASD) experience a deficit in facial emotion recognition. The dopaminergic pathway has been implicated in the pathogenesis of ASD. This study was aimed at determining facial emotion recognition and its correlation with polymorphisms in the dopaminergic pathway genes in children with ASD.

**Methods:**

Facial emotion recognition was examined in 98 children with ASD and 60 age- and gender-matched healthy controls. The severity of ASD was evaluated using the Childhood Autism Rating Scale (CARS). DNA from blood cells was used to analyze the genotypes of single-nucleotide polymorphisms (SNPs) in dopaminergic pathway genes. SNPs of DBH rs1611115, DDC rs6592961, DRD1 rs251937, DRD2 rs4630328, and DRD3 rs167771 were analyzed.

**Results:**

Children with ASD took a significantly longer time to recognize all facial emotions, and their interpretations were less accurate for anger at low intensity and fear at both low and high intensities. The severity of the disease was associated with significant delays in recognition of all facial emotions and with a decrease in accuracy in recognition of happiness and anger at low intensity. Accuracy in recognizing fear at high intensity and sadness at low intensity was associated with rs251937 and rs4630328, respectively, in children with ASD. Multivariate logistic regression analysis revealed that SNP rs167771, response time for the recognition of happiness, sadness and fear, and accuracy in recognition of anger and fear were all associated with the risk of childhood ASD.

**Conclusions:**

Children with ASD experience a deficit in facial emotion recognition. Certain SNPs in the dopaminergic pathway genes are associated with accuracy in recognizing selective facial emotions in children with ASD.

## 1. Background

Autism spectrum disorder (ASD) is a neurodevelopmental disorder characterized by impairment in social communication and by the presence of repetitive and restricted behaviors. Recognition of facial emotions is an important skill for normal social communication and interaction. Facial recognition is correlated with various neurological mechanisms related to visual face perception and/or memory [1]. Deficits in facial expression recognition ability can lead to impairment in social communication, which is the core symptom in patients with ASD [2, 3]. Interventions targeted toward the improvement of facial emotion recognition ability have been shown to enhance performance in social communication for children with ASD [4]. However, research has produced mixed findings. Some studies have reported generalized deficits in recognition of various emotions, and some have reported deficits only in the recognition of specific emotions [5, 6]. Other studies found no differences in facial emotion recognition between patients with ASD and healthy control subjects [7, 8].

The neurotransmitter dopamine plays an essential role in the coordination of body movement, motivation, and arousal [9, 10]. Dopamine is synthesized from DOPA by DOPA decarboxylase (DDC). Dopamine beta-hydroxylase (DBH) catalyzes the conversion of dopamine into norepinephrine [11]. The function of dopamine is mediated through its receptors. The dopamine receptor (DR) belongs to the G protein-coupled receptor family. At least five subtypes of DR have been identified so far [12]. DRD1 is the most abundant dopamine receptor in the central nervous system. The dopaminergic pathway has been implicated in the pathogenesis of ASD [13]. Abnormal dopamine activity in the brain and increased catecholamine levels in blood, urine, and cerebrospinal fluid have been found in some autistic children. Dysfunction of the dopaminergic pathway has been associated with many neurological and psychiatric diseases, such as Parkinson's disease, schizophrenia, and attention-deficit hyperactivity disorder (ADHD) [14].

The correlation between ASD and single-nucleotide polymorphisms (SNP) in dopaminergic pathway genes has been studied previously. Toma et al. found that SNP rs6592961 in the DDC gene was significantly associated with ASD in a European population and that DRD1 rs251937, DRD2 rs4630328, and DRD3 rs167771 were nominally associated with ASD in the additive model [15]. Hettinger et al. found that rs265981, rs4532, and rs686 in the DRD1 gene were significantly associated with autism in European and American populations [16]. de Krom et al. studied 144 Dutch and 128 British children with ASD and found that rs167771 of the DRD3 gene was significantly associated with ASD [17]. Further study in 91 patients with ASD revealed a significant association between the SNP s167771 and a specific type of repetitive behavior [18]. A marginal association between DBH rs161115 and ASD was observed from analyzing the overall dataset consisting of 403 non-Hispanic Caucasian American families [19]. In addition, overactivation of DRD1 in normal mice or the genetic knockout of DRD2 was shown to produce typical autistic-like behaviors in mice [20]. Maternal separation induced autistic-like behaviors in DRD2 heterozygous knockout mice, but not in wild-type mice [21].

A few studies have reported the correlation between social or emotional recognition skills and polymorphisms in the oxytocin receptor (OXTR) gene [22] and the aryl hydrocarbon receptor nuclear translocator 2 (ARNT2) gene [23] and COMT gene [13] in the general population. We hypothesized that deficits in facial emotion recognition were correlated with genetic polymorphisms in the dopaminergic pathway genes in children with ASD. This study was aimed at examining whether children with ASD had deficits in facial emotion recognition and whether these deficits were associated with polymorphisms in genes related to the dopaminergic pathway.

## 2. Methods

### 2.1. Participants

Han Chinese children affected with ASD were recruited from hospitals in Xiaoshan District of Zhejiang Province based on the criteria described previously [24]. Healthy control children were born of normal pregnancies and births with no history of physical or mental disorders and were recruited from schools in the same district. A total of 98 children with ASD and 60 age- and gender-matched healthy children were enrolled from May 2017 to June 2018. The average age was 7.9 ± 3.3 (range: 6-10) years for children with ASD and 8.1 ± 3.5 (range: 6-11) years for healthy control subjects (*P* = 0.7184). Males accounted for 78.3% and 81.6% of children with ASD and healthy controls, respectively. The severity of ASD was evaluated using the Childhood Autism Rating Scale (CARS). Children with scores of <36 were considered to have mild-moderate ASD (*n* = 37), and children with scores ≥ 36 were considered to be severely affected (*n* = 61). This study was approved by the Medical Ethics Committee of Zhejiang Xiaoshan Hospital. Informed consent was obtained from parents or guardians of all children.

### 2.2. Genotyping

SNPs DBH rs1611115, DDC rs6592961, DRD1 rs251937, DRD2 rs4630328, and DRD3 rs167771 were analyzed in this study. Information related to these SNPs is listed in Supplemental Table S1. DNA was extracted from blood cells and analyzed using a TaqMan probe-based PCR approach [24]. Both TaqMan probes and real-time PCR reagents were purchased from Applied Biosystems (Beijing, China). The catalog numbers are C___2535786_10 for rs1611115, C__29250259_10 for rs6592961, C___3199281_30 for rs251937, C__11339292_10 for rs4630328, and C____949778_10 for rs167771. Real-time PCR was conducted with the ABI7900 real-time PCR instrument following the manufacturer's protocol, described previously [24]. Briefly, the PCR reactions were performed in a total volume of 15 *μ*l containing 20 ng of genomic DNA, 0.4 *μ*l of the primers and probe mixture, 7.5 *μ*l of universal PCR mix, and water. PCR cycles used were as follows: 95°C for 10 minutes and then 40 cycles of 95°C for 15 seconds and 60°C for 1 minute. The results were analyzed using the ABI SDS2.3 software.

### 2.3. Assessment of Facial Emotion Recognition

A total of 16 pictures depicting facial expression associated with common emotions were selected for facial emotion recognition [25]. These pictures represented 4 emotions (happiness, sadness, anger, and fear) at low or high intensity. The experiment was divided into a practice stage and a formal experiment stage. Eight pictures were used for practice, and 8 different pictures for the formal experiment stage. Both practice and experiment were carried out on a computer in a quiet room in the presence of the parents. Pictures of facial emotions were presented in random order and remained on the screen while the participants chose a response.

### 2.4. Statistical Analysis

All data were analyzed using SAS 9.3 software (SAS Institute Inc., Cary, NC). The sample size was decided based upon similar studies reported previously [18, 25, 26]. Two-way ANOVAs were conducted to test the response time of facial emotion recognition between the two groups of subjects (children with ASD and heathy controls), between children with different levels of severity of ASD, and among children with different SNPs. Post hoc pairwise comparison was analyzed using the Tukey test. *χ*^2^ analysis was used to evaluate accuracy in facial emotion recognition, to examine the differences in SNP frequencies between children with ASD and healthy controls and between children with different levels of severity of ASD, and to analyze the data for the Hardy-Weinberg equilibrium. When over 20% of cells had expected counts < 5 in a *χ*^2^ test, Fisher's exact test was used to compute *P* values. The false discovery rate (FDR) approach was applied to adjust multiple comparisons. Multivariate logistic regression analysis was applied to identify factors associated with the risk of childhood ASD. Odds ratios (ORs) and 95% confidence intervals (CIs) were calculated. A significance level of *P* < 0.05 (two-sided) or FDR < 10% was adopted.

## 3. Results

### 3.1. Comparison of the Response Time for Facial Emotion Recognition

Our results showed that the response time for facial emotion recognition ranged from 5.8 to 8.9 seconds for children with ASD and from 2.9 to 7.2 seconds for healthy control subjects. Children with ASD responded significantly more slowly than the healthy controls in recognizing every facial emotion presented (Table 1). Children in both groups took a longer time to recognize low-intensity facial emotions than strong-intensity facial emotions. Based on the scores of CARS, children with ASD were divided into mild-moderate and severe groups. Children with severe ASD displayed significantly slower response times to every facial emotion presented, compared to children with mild-moderate ASD (Table 1).

### 3.2. Comparison of the Accuracy in Facial Emotion Recognition

Compared to healthy controls, children with ASD were significantly less accurate in recognition of anger at low intensity (*P* < 0.0001) and fear at both high (*P* = 0.0272) and low intensities (*P* = 0.0087) (Table 2). Among all facial emotions, children with ASD had the lowest accuracy (40.8%) in recognizing anger at low intensity, whereas healthy control subjects had the lowest accuracy in recognizing fear at low intensity (68.3%). Children with severe ASD were significantly less accurate in recognizing happiness at low intensity (*P* = 0.0493) and anger at low intensity (*P* = 0.0124), but not other facial emotions (Table 2).

### 3.3. Association between SNPs and the Risk of ASD

Genotypic distributions of SNPs rs1611115, rs167771, rs4630328, rs251937, and rs6592961 were all in accordance with the Hardy-Weinberg equilibrium for both groups (Supplemental Table S2). There was no significant difference in frequencies of these SNPs in children with ASD compared to healthy controls or in children with mild-moderate ASD compared to children with severe ASD (Table 3).

### 3.4. Association between SNPs and Facial Emotion Recognition

Our data revealed that no SNP was associated with response time for recognition of facial emotions in children with ASD (Supplemental Table S3). In contrast, SNP rs251937 was significantly associated with accuracy in recognition of fear at strong intensity, and rs4630328 was significantly associated with accuracy in recognition of sadness at low intensity in children with ASD (Table 4).

### 3.5. Risk Factors in Predicting the Risk of ASD

The potential roles of facial emotion recognition and SNPs in identifying children with ASD were examined using the multivariate logistic regression model. Our data showed that SNP rs167771; response time for recognition of happiness at high intensity, sadness at both low and high intensities, and fear at low intensity; and accuracy in recognition of anger at low intensity and fear at high intensity were all associated with the risk of childhood ASD (Table 5).

## 4. Discussion

Deficits in facial emotion recognition have been associated with impaired social communication in patients with ASD. Very few studies have investigated the correlation between facial emotion recognition and polymorphisms in patients with ASD. This study examined facial emotion recognition and SNPs in the dopaminergic pathway genes in children with ASD and healthy controls from the Han Chinese population. Our data revealed that children with ASD took a significantly longer time to recognize all facial emotions presented to them and they demonstrated a lower rate of accuracy in recognizing specific emotions. Children with severe ASD demonstrated worse performance in recognizing facial emotions. Though none of the examined SNPs was associated with response time for facial emotion recognition, the SNPs rs251937 and rs4630328 were significantly associated with accuracy in recognition of fear at strong intensity and sadness at low intensity, respectively, in children with ASD. SNP rs167771 (genotype G/G vs. AA), delayed response time, and decreased accuracy in recognizing specific facial emotions were all associated with an elevated risk of childhood ASD.

Our first major finding was that children with ASD demonstrated a decreased ability to recognize facial emotions. Among the four facial emotions (happiness, anger, sadness, and fear) examined, children with ASD took significantly more time to recognize all these emotions at both high and low intensities. They also demonstrated significantly lower accuracy in recognizing anger at low intensity and fear at both high and low intensities. Deficits in recognizing facial emotions in patients with ASD have been reported previously [25–27]. Teunisse and de Gelder reported that patients with ASD needed longer response times to recognize facial emotions [28]. Autistic patients were found to be less accurate in recognizing emotions at low intensities [25, 29, 30]. Griffiths et al. found that patients with ASD were less accurate in interpreting facial emotions across intensity levels [31]. Patients with ASD might have deficits in recognition of just some of the emotions [5, 28]. In contrast, other studies found no significant difference in response time and accuracy for facial emotion recognition between children with ASD and healthy controls [7, 32]. These mixed findings may be due to different age groups, ways of assessing facial emotion recognition, the severity of the disease, and populations used in different studies.

Previous studies have found that children with low social functioning demonstrated deficits in facial emotion recognition [2, 26]. Social functioning in children with ASD was not examined in our study. Instead, the severity of ASD was evaluated by CARS in the affected children in this study. Our data showed that children with severe ASD required significantly longer response times to recognize facial emotions and were less accurate in recognizing happiness and anger at lower intensities. This finding suggests that the severity of the disease has an impact on performance in recognizing facial emotions.

The dopaminergic pathway has been implicated in the etiology of ASD [33]. This study examined SNPs in dopaminergic pathway genes including DBH, DDC, and DRD1-3. In contrast to previous findings in other populations [15–17], the frequencies of SNPs were not significantly different between children with ASD and healthy controls and between children with mild-moderate ASD and children with severe ASD in this study. This finding is consistent with our previous study [24]. Additional studies using a larger number of subjects are needed to corroborate the correlation between these SNPs and childhood ASD in the Chinese population.

Facial emotion recognition is a heritable trait and therefore may be strongly influenced by genetic background. Indeed, social recognition skills were associated with polymorphisms in the OXTR gene in the general population [22]. Aryl hydrocarbon receptor nuclear translocator 2 (ARNT2) is a transcription factor participating in the development of hypothalamic oxytocin and vasopressin neurons. The SNP rs4778599 in the gene ARNT2 was shown to be associated with emotion recognition in women [23]. This study examined the association between polymorphisms in dopaminergic pathway genes and facial emotion recognition in patients with ASD. Our data revealed that SNPs in dopaminergic pathway genes were not associated with response time for facial emotion recognition in children with ASD. Two of the five SNPs were associated with accuracy in recognition of facial emotions.

ASD is known to be caused by a complex interplay of multiple susceptibility genes and environmental factors. First-degree relatives of autistic patients appear to share deficits in emotion recognition [34]. Deficits in facial emotion recognition have been considered for use as an endophenotype for ASD [35]. Facial emotion recognition may further serve to stratify children with ASD [36]. This study revealed that time required to recognize happiness and sadness at high intensity and sadness and fear at low intensity, accuracy in recognition of anger and fear at low intensity, and SNP rs167771 in the gene DRD3 were associated with the risk of childhood ASD. It is noted that face recognition is correlated with the temporal lobe or the fusiform face area in the brain [1] and DRD3 is preferentially overexpressed in the striatum [37, 38]. Previous studies reported that the SNP rs167771 was a risk factor for ASD [17] and a biomarker related to repetitive behaviors in ASD [18, 39]. However, the varied genotypes (A/A, G/G, and G/A) of the SNP rs167771 do not have an impact on the functions of DRD3 [40]. More studies are needed to determine the role of the SNP rs167771 in the development of ASD.

## 5. Conclusions

Our study shows that children with ASD display a deficit in facial emotion recognition and that accuracy in selective facial emotion recognition is associated with certain SNPs in dopaminergic pathway genes in children with ASD.

## Figures and Tables

**Table 1:** Comparison of the response time (seconds) for recognition of facial emotions between groups.

Facial expression	Intensity	Healthy control (*n* = 60)	Children with ASD (*n* = 98)	*P* value	Children with ASD		*P* value
					Mild-moderate (*n* = 37)	Severe (*n* = 61)	
Happiness	High	2.9 ± 1.4	5.8 ± 2.4	<0.001	4.5 ± 2.0	6.5 ± 2.3	<0.001
	Low	5.6 ± 1.9	7.5 ± 2.0	<0.001	6.3 ± 2.0	8.2 ± 1.7	<0.001
Sadness	High	5.4 ± 1.8	7.3 ± 2.0	<0.001	5.9 ± 1.4	8.1 ± 1.8	<0.001
	Low	6.6 ± 1.8	8.9 ± 2.0	<0.001	7.7 ± 2.1	9.6 ± 1.6	<0.001
Anger	High	6.7 ± 1.7	8.4 ± 2.2	<0.001	7.0 ± 2.0	9.2 ± 1.9	<0.001
	Low	7.2 ± 1.3	8.7 ± 2.1	<0.001	7.3 ± 2.0	9.5 ± 1.6	<0.001
Fear	High	4.8 ± 1.5	6.0 ± 1.4	<0.001	5.1 ± 1.2	6.5 ± 1.2	<0.001
	Low	5.9 ± 1.2	7.2 ± 1.7	<0.001	6.3 ± 1.7	7.6 ± 1.5	<0.001

**Table 2:** Comparison of accuracy (%) in facial emotion recognition.

Facial emotion	Intensity	Healthy control (*n* = 60)	Children with ASD (*n* = 98)	*P* value	Children with ASD		*P* value
					Mild-moderate (*n* = 37)	Severe (*n* = 61)	
Happiness	High	96.7	87.8	0.0557	94.6	83.6	0.1077
	Low	75.0	66.3	0.2499	78.4	59.0	0.0493
Sadness	High	88.3	84.7	0.5213	83.8	85.3	0.8455
	Low	75.0	64.3	0.1599	67.6	62.3	0.5975
Anger	High	80.0	74.5	0.4273	83.8	68.9	0.1002
	Low	81.7	40.8	<0.0001	56.8	31.1	0.0124
Fear	High	85.0	69.4	0.0272	73.0	67.2	0.5487
	Low	68.3	46.9	0.0087	51.4	44.3	0.4954

**Table 3:** Genotype frequencies of SNPs in children with ASD and in healthy controls.

SNP	Genotypes	Healthy control, *n* (%)	Children with ASD, *n* (%)	*P* value	Children with ASD		*P* value
					Mild-moderate, *n* (%)	Severe, *n* (%)	
rs1611115	C/C	38 (63.3)	52 (53.1)	0.4428	20 (54.1)	32 (52.5)	0.4007
	C/T	19 (31.7)	39 (39.8)		16 (43.2)	23 (37.7)	
	T/T	3 (5.0)	7 (7.1)		1 (2.7)	6 (9.8)	

rs6592961	G/G	43 (71.7)	63 (64.3)	0.6312	22 (59.5)	41 (67.2)	0.6838
	G/A	14 (23.3)	29 (29.6)		12 (32.4)	17 (27.9)	
	A/A	3 (5.0)	6 (6.1)		3 (8.1)	3 (4.9)	

rs251937	C/C	15 (25.0)	15 (15.3)	0.3084	6 (16.2)	9 (14.8)	0.8049
	C/T	32 (53.3)	57 (58.1)		20 (54.0)	37 (60.7)	
	T/T	13 (21.7)	26 (26.5)		11 (37.8)	15 (24.6)	

rs4630328	G/G	57 (95.0)	92 (93.9)	0.7677	35 (94.6)	57 (93.4)	0.8176
	G/A	3 (5.0)	6 (6.1)		2 (5.4)	4 (6.6)	

rs167771	A/A	36 (60.0)	72 (73.4)	0.1772	25 (67.6)	47 (77.0)	0.5593
	G/A	20 (33.3)	21 (21.5)		10 (27.0)	11 (18.0)	
	G/G	4 (6.7)	5 (5.1)		2 (5.4)	3 (4.9)	

**Table 4:** Association between SNPs and accuracy in facial emotion recognition in children with ASD.

Facial expression		Happiness		Sadness		Anger		Fear	
Intensity		High	Low	High	Low	High	Low	High	Low
rs1611115	C/C	82.7	61.5	84.6	59.6	76.9	46.2	69.2	48.1
	T/C	92.3	74.4	82.5	66.7	66.7	33.3	66.7	46.2
	T/T	100	57.1	100.0	85.7	100.0	42.9	85.7	42.9

rs6592961	A/A	100.0	66.7	66.7	66.7	66.7	66.7	66.7	66.7
	G/A	92.1	69.0	86.2	62.1	75.9	34.5	55.2	41.4
	G/G	84.1	65.1	85.7	65.1	74.6	41.3	76.2	47.6

rs251937	C/C	100	80.0	100	73.3	86.7	40.0	*86.7*	26.7
	T/C	82.5	64.9	80.7	59.7	66.7	31.6	*54.3*	42.1
	T/T	92.3	61.5	84.6	69.2	84.6	61.5	*92.3*	69.2

rs4630328	G/A	66.7	66.7	66.7	*0*	33.3	33.3	33.3	33.3
	G/G	83.7	66.3	85.9	*68.5*	77.2	41.3	71.7	47.8

rs167771	A/A	86.2	63.8	86.2	62.1	70.7	34.5	67.2	46.6
	G/A	87.1	74.2	83.9	67.7	80.6	51.6	77.4	51.6
	G/G	100	55.6	77.8	66.7	77.8	44.4	55.6	33.3

The numbers in italics indicate that accuracy in facial expression recognition was significantly different for these SNP variants (FDR < 5%).

**Table 5:** Multivariate analysis of predictive factors associated with the risk of childhood ASD.

Variable	Odds ratio (95% CI)	*P* value
rs167771		
G/A vs. A/A	9.1 (2.1-39.6)	0.0034
G/G vs. A/A	1.0 (0.1-7.5)	0.9688
Time to recognize happiness at high intensity	3.0 (1.8-4.8)	<0.0001
Time to recognize sadness at high intensity	1.9 (1.2-2.8)	0.0028
Time to recognize sadness at low intensity	1.6 (1.2-2.3)	0.0022
Time to recognize fear at low intensity	2.0 (1.3-3.2)	0.0037
Accuracy in recognition of anger at low intensity (correct vs. incorrect)	0.1 (0.03-0.5)	0.0054
Accuracy in recognition of fear at high intensity (correct vs. incorrect)	0.04 (0.01-0.3)	0.0015

CI: confidence interval.

## Data Availability

The datasets used during the current study are available from the corresponding author on reasonable request.

## Abbreviations

ARNT2: Aryl hydrocarbon receptor nuclear translocator 2
ASD: Autism spectrum disorder
COMT: Catechol-O-methyltransferase
DBH: Dopamine *β*-hydroxylase
DDC: DOPA decarboxylase
DRD: Dopamine receptor D
OXTR: Oxytocin receptor.

## Supplementary Materials

Supplementary Materials Supplemental Table S1: detailed information for the examined SNPs. Supplemental Table S2: Hardy-Weinberg equilibrium for SNPs. Supplemental Table S3: association between SNPs and response time for facial emotion recognition in children with ASD.
