# Supplementary material for: Facial Emotion Recognition and Polymorphisms of Dopaminergic Pathway Genes in Children with ASD
Source: Behav Neurol. 2020 Nov 4;2020:6376842. doi: 10.1155/2020/6376842 (PMC7657692; doi:10.1155/2020/6376842)
Supplement: Supplementary Materials — Supplemental Table S1: detailed information for the examined SNPs. Supplemental Table S2: Hardy-Weinberg equilibrium for SNPs. Supplemental Table S3: association between SNPs and response time for facial emotion recognition in children with ASD. [file 6376842.f1.docx]

Supplemental Description

Supplemental Table S1. Detailed information for the examined SNPs

Supplemental Table S2. Hardy-Weinberg equilibrium for SNPs

Supplemental Table S3. Association between SNPs and response time for facial emotion recognition in children with ASD
